# A Qualitative Study of Center Director, Teacher, and Parent Input for Delivering a Virtual Early Childhood Mental Health Consultation Model in the Aftermath of COVID-19

**DOI:** 10.1007/s10643-024-01809-3

**Published:** 2024-12-24

**Authors:** Sara M. St. George, Elizabeth Howe, Carolina Velasquez, Anais Iglesias, Tomilola T. Awojobi, Yaray Agosto, Alejandra Casas, Rebecca J. Bulotsky-Shearer, Jason F. Jent, Ruby A. Natale

**Affiliations:** 1 Department of Public Health Sciences, University of Miami Miller School of Medicine, 1120 NW 14th Street, Suite 1009, Miami, FL 33136 USA; 2 Department of Pediatrics, Mailman Center for Child Development, University of Miami Miller School of Medicine, Miami, FL USA; 3 Department of Psychology, University of Miami, Coral Gables, FL USA; 4 Department of Epidemiology, Robert Stempel College of Public Health & Social Work, Florida International University, Miami, FL USA

**Keywords:** COVID-19, Early childhood mental health consultation, Adaptation, Qualitative

## Abstract

Given disruptions to early care and education following the COVID-19 pandemic, it is important to mitigate long-term impacts of the pandemic on child development among ethnic and racial minority children. Our team is implementing an early childhood mental health consultation (ECMHC) model, or a multi-tiered intervention to support young children’s social-emotional development, that utilizes mental health consultants to deliver a virtual toolkit to ethnically and racially diverse early care and education centers. Understanding the perspectives and ongoing needs of center directors, teachers, and parents is critical to intervention delivery. Between February and April 2023, 18 participants (*n* = 6 center directors, *n* = 6 teachers, *n* = 6 parents) across 12 early childcare centers completed individual interviews in English or Spanish. We used a rapid qualitative analysis to generate four themes related to participants’ perceived impact of COVID-19, including how it (1) exacerbated existing financial and administrative challenges, (2) increased their need for adaptability, (3) highlighted the importance of support for staff facing educational challenges during a public health emergency, and (4) highlighted the value of partnerships between parents and centers. We generated five additional themes specific to participants’ ongoing needs and suggestions, including (1) increased financial support, (2) outside behavioral support, (3) enhanced center staff self-care, (4) balancing in-person interaction with planned virtual delivery, and (5) use of existing smartphone applications for communication with parents. In addition to informing adaptations to our model, including expanding upon program pillars (e.g., expanding our safety planning pillar to include financial safety via linkages to community resources), these data may be used to inform the delivery of other ECMHC programs for diverse populations.

## Introduction

High quality early care and education contributes to the academic, behavioral, and social-emotional development of children (Von Suchodoletz et al., 2023). Early care and education centers that offer safe environments, stable and nurturing relationships, and high-quality education not only provide a buffer against adverse childhood experiences but also foster the resiliency and adaptive skills necessary for academic readiness and future overall success (Berry et al., 2016; Sciaraffa et al., 2018). High quality early care and education are especially important for the development of young children from low-income, ethnically/racially diverse families given their higher likelihood of exposure to environmental risk factors (Smith, 2020). However, early care and education centers have been expected to prioritize the provision of high-quality services while facing ever increasing challenges (e.g., teacher turnover; Doromal et al., 2022), many of which were exacerbated by the COVID-19 pandemic (Carson & Mattingly, 2020).

Prior research consistently highlights associations between early care and education staff and teacher mental health and well-being and young children’s learning and development (Jeon et al., 2019; Sandilos et al., 2018; Whitaker et al., 2015). COVID-19 and its resulting policies (e.g., mandated closures) severely disrupted the early care and education industry, presenting center directors, teachers, parents, and children with unprecedented stressors (Crouse et al., 2023; Quinn et al., 2022). The Center directors navigated evolving federal, state, and local guidelines, higher costs associated with enhanced safety protocols, reduced revenue due to lower enrollment, and teacher and staffing shortages (Hoffman & Poll, 2022). Teachers faced issues such as abrupt changes in teaching modality with limited training and technological support, increased job responsibilities, financial hardships, and fear of infection (Ford et al., 2021; Kwon et al., 2022). They reported experiencing physical and behavioral symptoms of stress at rates two to three times higher than before the pandemic (Swigonski et al., 2021). Notably, early care and education staff and teachers from ethnic and racial minority backgrounds reported high stress, emotional challenges, poor sleep, and low quality of life (Souto-Manning & Melvin, 2022). Parents of young children also reported experiencing poorer mental health during this time (Racine et al., 2022) as they dealt with increased childcare demands and reduced support while fulfilling work and household duties (Dawes et al., 2021). Ultimately, the confluence of these stressors created downstream negative effects on the social-emotional development and learning experiences of young children, particularly those from low-income or ethnic/racial minority backgrounds (González et al., 2022; Jarvers et al., 2023; Liu et al., 2022; Robertson et al., 2021; Watts & Pattnaik, 2022). It is thus critical that interventions to support young children’s learning and development consider strategies related to the stress and well-being of diverse early care education staff, teachers, and parents in the aftermath of the pandemic.

The Georgetown Model of Early Childhood Mental Health Consultation (ECMHC) is an evidence-informed (e.g., Natale et al., 2022) multi-level model to promote positive social and emotional development within early care and education centers (Davis & Perry, 2015; Hunter et al., 2016). ECMHC was used prior to the pandemic as a source of support for childcare teachers. ECMHC is delivered by mental health consultants whose role is to develop collaborative relationships with early care and education directors and teachers to support positive outcomes in young children’s social and emotional development by teaching pro-social skills to prevent challenging behaviors. ECMHC programs vary in their implementation to provide individualized services that meet the needs of different programs, providers, and populations (Hunter et al., 2016). Outcomes from the implementation of ECMHC include improvements in children’s social skills and child/teacher relationships as well as reductions in suspensions and expulsions and providers’ stress, burnout, and turnover (The Center of Excellence for Infant & Early Childhood Mental Health Consultation, n.d.).

The Jump Start Early Childhood and Consultation program is an example of an ECMHC program adapted from the Georgetown University model with a specific focus on ethnically and racially diverse early childhood providers, young children and their families (Duran et al., 2010). During the COVID-19 pandemic, our team developed a mental health toolkit, Jump Start +: COVID-19 Support (JS + CS), to support childcare centers and prepare them for future potential public health outbreaks. JS + CS utilizes mental health consultants to deliver a virtual toolkit which addresses four program pillars (i.e., safety planning, communication, self-care, and behavior support). Our team is currently conducting a Type 1 hybrid effectiveness-implementation trial with 24 centers in South Florida, a prior epicenter of the COVID-19 pandemic. The goals of our trial are to evaluate the effects of the JS + CS program delivered via Kubi telepresence robots on child psychosocial functioning, teacher psychosocial functioning and classroom practices, and the center’s capacity to maintain safety and psychosocial support (Natale et al., 2023). The Kubi telepresence approach uses a stationary desktop robot with a tablet connected to secure video conferencing software that can pan and tilt, allowing remote users to control their view and participate more naturally in remote interactions with early care and education centers.

The impacts of COVID-19 on early care and education programs are still unfolding, and research points to the importance of attending to the health and well-being of ethnically and racially diverse early care education center staff and teachers given the significant impact of pandemic stressors on their quality of life (Souto-Manning & Melvin, 2022). In anticipation of emerging challenges, there is a critical need to understand the comprehensive multi-level support that childcare centers require to address COVID-19 impacts on center directors, teachers, parents, and children. Obtaining qualitative information allows us to better understand the needs of our unique population as a result of COVID-19 and inform necessary adaptations to ECMHC. As such, an important initial step in the process of implementing JS + CS for the trial was developing a rich understanding of the perspectives of diverse center directors, teachers, and parents regarding their experiences with and ongoing needs following the COVID-19 pandemic. These insights may also be used more broadly to inform the implementation of ECMHC programs for diverse populations. As such, the purpose of this qualitative study is to answer the following research questions: (1) How do center directors, teachers, and parents of young children from ethnic and racial minority backgrounds describe the impact of COVID-19 on early care and education? (2) How do they describe their needs related to the delivery of a virtual ECMHC model?

## Methods

### Participants and Setting

We purposively sampled participants (*n* = 18) in this study from 12 early care and education centers in Miami-Dade and Broward Counties participating in our Type 1 hybrid effectiveness-implementation trial (Natale et al., 2023). To be included, centers had to: have 50 or more children, of whom at least 30 were 18 months to three years of age; be located in a low-income census tract with at least 50% of families receiving childcare subsidies; and serve at least 60% Hispanic or 60% Non-Hispanic Black families. Notably, all but one of the 12 centers were in Miami-Dade County, the largest major metropolitan area in the state of Florida, with 2.7 million residents, including 54% who are foreign-born, 75% who speak a language other than English in the home, and 16% living below the federal poverty level (Florida Department of Health in Miami-Dade County, 2022). At the height of the COVID-19 pandemic, Miami-Dade County was identified as a COVID-19 hot spot (Allen, 2020; Nazaryan & Ramirez, 2020). In March 2020, 20% of the 1343 childcare programs in the county remained open, and unlike some states, 40% were open by May 2020 (Ocasio, 2020; Wolford, 2020). Five of the participating centers were randomly assigned to the JS + CS condition; seven centers were randomly assigned to the comparison condition.

Participants were eligible for this study if they were either a director, teacher, or parent of a child in a center enrolled in the trial. They were ineligible if they did not speak English or Spanish. To recruit participants, our team distributed flyers to teachers and parents and used a master list of participants in the trial to randomly select interviewees across three levels (directors, teachers, parents). We contacted potential participants in the order in which they appeared on our randomly generated list until we scheduled three interviews for English and Spanish speaking participants, respectively, across all levels. Sixty-one percent of participants were Hispanic and 83% had attained at least some college (see Table 1 for participant demographic characteristics).

**Table 1:** Participant demographic characteristics

Characteristic	Directors (*n* = 6)	Teachers (*n* = 6)	Parents (*N* = 6)
**Age**,** M**	52.9	49.7	33.8
**Years’ Experience**,** M**	18.8	16.2	–
**Language**,** n (%)**			
English	3 (50.0)	3 (50.0)	3 (50.0)
Spanish	3 (50.0)	3 (50.0)	3 (50.0)
**Race**,** n (%)**			
White	5 (83.3)	4 (66.7)	3 (50.0)
Black	0	2 (33.3)	3 (50.0)
Other	1 (16.7)	0	0
**Ethnicity**,** n (%)**			
Hispanic	5 (83.3)	3 (50.0)	3 (50.0)
Non-Hispanic White	0	1 (16.7)	0
Non-Hispanic Black	1 (16.7)	0	3 (50.0)
Haitian	0	2 (33.3)	0
**Education**,** n (%)**			
Some High School	0	0	1 (16.7)
High School/GED	1 (16.7)	1 (16.7)	0
Some College	1 (16.7)	3 (50.0)	1 (16.7)
Associate Degree	0	0	1 (16.7)
Bachelor’s Degree	2 (33.3)	2 (33.3)	3 (50.0)
Graduate Degree	2 (33.3)	0	0

## Procedures

This study was approved by the University of Miami Institutional Review Board. We conducted informed consent procedures during the trial onboarding process for each participating center and commenced recruitment for qualitative interviews once half the centers were onboarded. At the time of consent, participants agreed to be contacted for qualitative interviews. We compensated participants with Publix or Walmart gift cards for completing baselines surveys (center directors and teachers: $40; parents: $20) but not specifically for participating in interviews.

We developed a semi-structured interview guide (see Table 2). The interview guide was built around the domains of interest for the study including participants’ experience during and following the pandemic and areas of support for implementing a virtual ECMHC model. Examples of questions included, *“Tell me what COVID-19 was like for you as a (director*,* teacher*,* parent)”* and *“How do you think childcare centers should support (teachers*,* parents) in the areas of self-care*,* behavioral support*,* safety*,* communication*,* nutrition*,* and physical activity?”*

**Table 2:** Qualitative interview guides

Questions and Follow-Up Probes	Directors	Teachers	Parents
1. Tell me about what COVID-19 has been like for you as a [director, teacher, parent].	×	×	×
2. Tell me about the impact COVID-19 has had on your [center/family].	×	×	×
3. What guidance have you received related to COVID-19 [from your center]?	×	×	
4. What guidance have you received from your child’s center about their COVID-19 policies?			×
5. Tell me about your experience implementing these policies [in your classroom].	×	×	
6. Tell me about the impact COVID-19 has had on your experience with your child’s center?			×
7. What did [your/your child’s] center do to address the following *during the pandemic*: a. Self-care (staff support and supervision)? b. Behavior supports (teacher-child relationships, structured environment, social-emotional program practices)? c. Safety (physical safety and disaster recovery procedures)? d. Communication (teacher-parent communication and connection)? e. Nutrition? f. Physical activity?	×	×	×
8. What do you think directors need *now* to feel supported in these areas (within reason)?	×		
9. How do you think childcare centers should support teachers *now* in these areas (within reason)?		×	
10. How helpful do you view teleconsultations/tele-workshops vs. receiving in-person consultations/workshops? Why?	×	×	×
11. Do you think an app on your phone would be helpful to support you in identifying resources in these areas?	×	×	×

Graduate students in public health trained by the first author conducted interviews (*n* = 6 directors, *n* = 6 teachers, *n* = 6 parents) between March and April of 2023 using the Zoom videoconferencing platform. Interviews lasted on average M = 27.0, SD = 7.6 min. We recorded all interviews via Zoom and transferred recordings to a HIPPA-compliant University cloud storage system. Interviewers downloaded and checked the English language transcripts produced by Zoom to ensure accuracy. Bilingual interviewers transcribed interviews conducted in Spanish. We analyzed all transcripts in their original language.

## Qualitative Analysis

We used rapid qualitative analysis (RQA; Hamilton, 2013, 2020; Hamilton & Finley, 2019) to swiftly produce findings that would inform the adaptation of our virtual ECMHC model. RQA is a method of rigorously expediting qualitative analysis to address pressing issues, such as the timely adaptation of an intervention for ethnically and racially diverse children and families (St. George et al., 2023). The process involves summarizing interview transcripts using a standard template and subsequently transferring key points into a matrix used to explore relevant themes.

The first author trained five research team members (three of whom were fluent in English and Spanish) on RQA using a combination of didactic presentations on the relevance of RQA and its procedures and hands-on practice (i.e., populating an interview summary template and receiving feedback to ensure consistency and accuracy). The team then followed a structured process that involved two individuals reviewing each transcript. The first team member completed the initial interview summary, which is a 2-page document that summarizes key points for each question. The second team member then audited the interview summary for accuracy and transferred key statements into a matrix that contained all participant responses for each interview question. The first author used the matrix to generate preliminary themes, which were discussed and refined by the larger research team, including study PIs, Co-PIs, interviewers, and staff.

## Results

### Aim 1: The Impact of COVID-19 on Early Childhood Education

We generated four themes related to participants’ perceived impact of COVID-19, including how it (1) exacerbated existing financial instability and administrative challenges, (2) increased their need for adaptability across multiple domains (e.g., center-wide safety and health policies, classroom management, parent communication), (3) highlighted the importance of support for staff facing challenges during a public health emergency, and (4) highlighted the value of partnerships between parents and centers. We present each of these themes and illustrative quotes in the text below and supplemental quotes in Table 3.

**Table 3:** Themes and additional participant quotes

Theme	Sample Quotes
Aim 1: The Impact of COVID-19 on Early Childhood Education	
1. Exacerbated financial instability and administrative challenges	• “Financially, the costs went up, the teacher’s salaries also had to go up, the cost of living is higher, and we have seen that it affected not only our lives but also the business.” **–** ***Center Director*** • “For example, we tried when the first closure, which they said was expected to be for 15 days, we tried to cover a week’s salary for the teachers, but the next week we couldn’t do it.” **–*Center Director***
2. Increased need for adaptability Center-wide safety and health policies Classroom management practices Parent communication strategies	• “When we arrived in the morning, cleaning with water and chlorine. Before leaving we left everything ready again, disinfected with Lysol and everyone wore gloves. No one could take off their gloves, [they had to] wash their hands and put gloves back on. [We were] constantly washing the children’s hands.” - ***Center Director*** • “We have the new policy and procedures set up for us how to manage the class, how to prepare the classroom for kids to come back. The parents were not allowed to come to the classroom we have to meet them in front of the door. And what else, we taught the children to read the stories about the COVID, how to approach, and what that means.” -***Teacher*** • The teachers and directors themselves gave us information, they gave us [information about] how to make sure that if, as I said, [a child] had any discomfort or something, it was obviously to go to your pediatrician, do the COVID tests, ask your questions.” ***– Parent*** • “They invited us to many meetings like this through meetings like this via Zoom… to give us talks… about behavior, COVID, for how parents should be with children, so all those things.” ***– Parent***
3. Highlighted the importance of support for early care and education staff facing challenges during a public health emergency	• “Well, I know that I’m more stressful than ever, but I have to cope with it. I mean, I took a lot of classes that Miami-Dade offered for directors and coping with stressful situations.” - ***Center Director*** • “Yes, we communicate. We have little meetings, and we communicate with each other, you know, and then they usually offer us COVID tests, and we have the COVID center not too far…. And then we just like we’re looking out for each other.” -***Teacher***
4. Highlighted the value of partnerships between parents and centers	• “They were tragic days, and really, even at this point, well, I mean, it is something that changed our lives a lot. It affected us a lot and well that was the reason why I emigrated from my country. I came to the United States. My child was a small baby…. [his father] died when my child was only 11 months old. I really am very grateful for the care that I have for my child [at the center].” ***– Parent*** • “This one, we were closed for a month and a half, and after a month and a half we opened with the food program where families went and looked for the “Grab and go” food packages, something like that, I think they called it.” ***– Center Director***
**Aim 2: The Need for a Virtual Early Childhood Consultation Model**	
1. Increased financial support	• “Yes and there are times we have to take it out of their pockets. Because in reality [teachers have] the lowest pay there is, because you take care of children you have to like it, but also the economy is very bad.” ***– Center Director*** • “This is a very hard job, that is, it is a good job that requires a lot of dedication for the teachers to achieve, and I liked that you have included them in the recognitions, you have given them your gift card, and even if it is little $20, $25, I don’t know $50, I don’t remember how much, that was an encouragement for them, they were very happy. I have seen this since the pandemic, when early childhood education teachers were relieved of their duties, now they see us as front-line personnel who are exposed to everyone. - ***Center Director***
2. Outside behavioral support	• “I think that sometimes they demand a lot from us teachers and they don’t realize that we have to do so much… that sometimes we need that extra little break from outside help to come and see. Because children sometimes get tired of us. You talk to them and it’s like this again. But when someone different comes, it’s like a party” ***– Center Director*** • “I think that figure, the coach gets into the classroom with them, helps the teacher, and when they are doing things [the coach] even models how they can do it or also tells them when you are there watching the day-to-day because the coach also creates a much more empathetic bond with the teacher because one classroom is not the same as another, that is, you are going to attend to the exact reality of that teacher in her scenario. And it seems to me that that is like the tool, you are within the teacher’s natural space, you are with her children, you are watching and you are on her schedule and you are helping her, you are teaching, in the moment.”– ***Center Director***
3. Enhanced center staff self-care	• That’s important, because sometimes that’s as teachers, we forget to even drink water. What else? Like, encouragement, affirmations– ***Teacher*** • You know I feel like moving for yoga, especially yoga. It gives you like self-control.– ***Teacher*** • I also would like to develop a system where my teachers can go at least another lunch hour, at least for like 15 min for a break, a coffee break. Because it’s a lot of work, a lot of attention, a lot of stress. And I don’t have that. We work 12 h. They only get that hour lunch from 12:30 to 1:30, and that’s it. I would like to implement, I’m trying to implement that, that rotation system, the way they can go out to the lounge they have, like, maybe a coffee or something like that.– ***Center Director***
4. Balancing in-person interaction with planned virtual delivery	• “So this has also helped, Zoom has really helped us to have that balance, to feel a little more comfortable, to use the time that was not used before, such as lunches. So now when they ask us what time we can do such a thing [workshops], at 12:30 or 1:00, which is when the little children are sleeping and one is here, you know, doing, well, there is a lot to do, we have to plan and everything, but it’s something that the work day is over and I can go and relax, I don’t have to be anywhere at 6:00 or 7:00 at night.” *–****Center Director*** • “I like them more in person with the parents because well, I think there is more connection with the person, I feel that it is a space of greater confidentiality for both the parent and us. Sometimes they take, they address topics that are not easy, and it is not the same to send it in a written text or say it from a screen, than to be sitting with the parent and well…I feel that in person, well, for me personally. I like it better in person. I still use Zoom but I use it when, for example, a parent tells me I don’t have time, well Zoom is better for you, well let’s do it by zoom ok. But generally I look for it to be in person…” ***– Center Director***
5. Use of existing smartphone applications for communication with parents	• “We started using Class Dojo, and we use it to communicate with the parents, sending the activities and lessons we are doing.” ***– Teacher*** • “We have an application where we send communication to parents daily. We worked with BrightWheel, at the time we worked with ClassDojo and you know we kept them up to date.” ***– Center Director***

### Theme 1: Exacerbated Financial Instability and Administrative Challenges

Participants described how the COVID-19 pandemic and community mitigation efforts exacerbated existing financial and infrastructural instability of early care and education. Directors and teachers reported that center closures early in the pandemic led to decreases in student enrollment. One director noted, “It’s been hell, because financially, no students, they don’t come to school like they should. We’ve had to shut down and just trying to keep everything clean. It’s been really hard.” Another added, “…from a business point of view it was very distressing, because well, even though we were closed, you still had to pay the rent, pay all the electricity, water, and insurance services, everything.” When centers were able to open, they noted that restrictions on class sizes led to substantial income loss. Directors also described increased staff turnover and teacher shortages, noting “good teachers did not return to work” and acknowledging the role of recent cost-of-living increases on historically underpaid staff.

### Theme 2: Increased Need for Adaptability

Directors described their need to follow continuously changing federal and state mitigation policies and their sense of urgency to implement them “immediately.” They reported that teachers generally accepted and applied the policies more willingly than parents, with some noting parents were “very responsible” and others noting “parents did not want to comply.” Participants described how challenges associated with COVID-19 increased the need for adaptability within early care and education across multiple domains, including center-wide safety and health policies, classroom management practices, and parent communication strategies.

#### Center-wide Safety and Health Policies

Directors and teachers described their implementation of physical safety procedures, including the use of personal protective equipment (e.g., gloves, masks, shields), the regular cleaning/sanitation of toys and other classroom materials, hand-washing, social distancing, regular COVID-19 testing, and heightened vigilance during this time. One director noted the following:

We have to, to be close supervision in everything and also if we see, observe a child that is coughing or runny nose, any symptoms related to COVID, we need to be aware and call the parents to let them know, the, the situation, how we observe the child.

Parents also described their need to adapt to evolving safety and health polices and lamented their restricted access to their children’s classrooms:

It was like, no, due to COVID we’re not allowing anyone back there. And I’m like, oh man, so the first years these milestones that my child is reaching I’m unable to participate and engage in it due to the impact of COVID.

Another parent added, “I’m unable to be involved. And, you know, hands on as I would like to be…”.

#### Classroom Management Practices

Regarding classroom management practices, teachers recounted how they separated children inside the classrooms for social distancing and planned activities with these adaptations in mind. One teacher noted:

When they come in, we have our dividers, because, of course, they have to be separated six feet. I had like for my kids, I have them coming in, I had rulers and I measured it. So, you know, six feet away from each other. So, it’s easy for them to understand, okay, this is six feet.

Teachers also described the integration of COVID-19-related content into lesson plans to support children’s understanding about policy changes (e.g., reading books about wearing masks). For example, “We taught the children to read the stories about the COVID, how to approach, and what that means.” Teachers and directors additionally shared changes they made to outdoor play (e.g., dividing playgrounds spatially to avoid interactions with children from other classrooms) and mealtimes (e.g., serving meals individually inside classrooms rather than in cafeteria area).

#### Parent Communication Strategies

Considering parents’ restricted access to classrooms, directors reported that teachers used different modes of technology to communicate with parents. One director described teachers’ use of existing messaging applications:

Every teacher developed a WhatsApp group with the parents, and they were in constant communication. Even the children who didn’t come because they had COVID, they still got, they were able to Zoom with the teachers and do the class and not miss the material.

Teachers also reported using apps such as Brightwheel™ (BrightwheelTM, 2023) and ClassDojo (ClassDojo Inc. ©, n.d.), a virtual classroom management platform and application that allows teachers to record student behavior and engage in home-school communication. They described using this app to provide parents with lesson plans and activities to support their child’s development.

### Theme 3: Highlighted the importance of Support for Early Care and Education Staff Facing Challenges During a Public Health Emergency

Directors and teachers used words such as “rough”, “hell”, “overwhelming”, and “chaos” to describe their experiences during the COVID-19 pandemic. One director summarized the experience as follows:

[The pandemic was] extremely challenging, extremely challenging. We’re not only with the children getting sick, parents getting upset that we don’t let them come in anymore inside the school and my teachers getting sick… I’ve been closing down classrooms very often, never before, and our enrollment dropped dramatically like a lot. So yes, very challenging.

Teachers reported difficult experiences using the Zoom videoconferencing platform to provide early childhood curriculum content to toddlers due to their age: “How to approach them and the zoom conversation we ended up with making the class, zoom class where the 3-year-olds and after 15 minutes we realized that we talking to the parents not the kids.” They also described numerous challenges they encountered when programs re-opened (e.g., having to keep children apart, explain social distancing to toddlers, restrict the number of toys in the classroom, plan activities in compliance with social-distancing policies) and worrying about their students and their families when they were absent. For example, one teacher commented:

So, everything was very calculated. I mean, there were a few toys. We had to do other types of activities and crafts with them… Wearing their masks, really, it was a bit difficult in that part and how to get them to get involved again.

Despite these challenges, teachers described the communication from center administrators as helpful and supportive:

They always were coming to the centers and show us, you know, examples or tell us if it’s okay or if it’s not okay. And no, I don’t feel that it was difficult. And it was like they made them so simple to understand that, that I didn’t have to read 10 pages of something. I just can send an email, and they respond right away about problems that I can face.

Communication occurred either through meetings or emails and this communication also included how teachers should consider their own self-care and use of personal protective equipment. Furthermore, directors reported that support and guidance from local and national government agencies, such as the Early Learning Coalition of Miami-Dade/Monroe Counties, the Department of Education, the Florida Department of Children and Families and the Centers for Disease Control and Prevention were helpful in implementing policies and communicating with teachers.

### Theme 4: Highlighted the value of partnerships between parents and centers

Parents described the early period of the pandemic as extremely stressful, using words such as “extremely”, “very”, “super-duper” hard, and a “high stress environment.” One parent said that “this COVID [interview] is like, oh my God, it’s bringing back horrible memories.” This period resulted in financial challenges for parents, with one single mother reporting living in a shelter with her three children under the age of 3 during the early stages of the pandemic and another noting COVID-19 “financially demolished” them. Another mother shared that she lost her significant other to COVID-19 when her son was 11-months old and migrated to the United States on her own. Overall, parents reported high stress: “Parents and individuals’ period, you know need breaks. So, unfortunately, I wasn’t able to get the break and support I needed.”

Yet despite these heightened levels of stress and reductions in face-to-face communication with teachers, most parents reported being satisfied with their communication with teachers and early care and education staff. Parents generally described being happy with their program and child’s teachers and thought they were doing a good job staying connected: “I mean, really, in the care that I received for my child, in that care, I am really grateful, I am really super happy in every sense with all the teachers, with the guidance, really in everything.” Though there was one notable exception of a parent who felt the program was traumatizing her son by making him feel they were all going to die from the virus:

Even when, after the mask mandate was over, [my son] continued to wear his mask because he was afraid to die, and I feel like the whole pandemic was very traumatizing to him, and the school did not do enough to you know, reinforce, like to secure the kids or make them feel safe or make them feel like, you know, they weren’t going to die from COVID.

Others reported receiving resources and support from centers, such as handouts and other types of information about COVID-19, the steps they could take if their child became ill from the virus, resources for financial support, resources for mental health for themselves, and virtual therapy, along with a general sense of love, care, and encouragement from teachers. Directors additionally described their efforts and role in food distribution to families when centers were closed. Overwhelmingly, parents indicated that they wanted to be more involved in their child’s early care and education center, especially to meet other parents.

### Aim 2: The Need for a Virtual Early Childhood Consultation Model

We generated five additional themes specific to participants’ ongoing needs and suggestions, including (1) increased financial support, (2) outside behavioral support, (3) enhanced center staff self-care, (4) balancing in-person interaction with planned virtual delivery, and (5) use of existing smartphone applications for communication with parents.

### Theme 1: Increased Financial Support

Participants noted that the need for increased financial support remains following the pandemic, especially with an inflated economy. One parent stated, “It’s the finances that like the inflation of everything. It’s just really out of hand. That’s the area, the focal point.” Directors described the need to increase teacher salaries to offset teacher shortages, with one noting “teachers get paid too little.” They also described the need for more money to buy supplies for teachers and materials for children. Directors additionally indicated that the financial support they received from the university for their participation in the trial was appreciated and helpful: “The monetary support from Jump Start [gift cards] are very appreciated; these are great incentives for teachers and staff to continue their work.”

### Theme 2: Outside Behavioral Support

Teachers provided examples, notably few, of behavioral and social-emotional strategies they implemented in their classrooms, including doing yoga with children, using a reward system when children followed directions, and having children label emotions. They described using brochures to outside behavioral analysis intervention agencies for families to contact if they had concerns and making referrals for children as needed: “We, that have like list of behavioral therapists, you know, ABA. Yeah. ABA. So, for the kids that have behavior issues, we have a lot of those in our classes now.” Directors shared that they would like teachers to have coaches from outside organizations in their classrooms to help support lesson planning, activities for children, and help with children who are demonstrating behavioral challenges:

The coach gets into the room with them, helps the teacher, and when they are doing things, even models how they can do it or also tells them when you are there watching the day to day… And it seems to me that that is like the tool, you are within the teacher’s natural space, you are with her children, you are watching and you are on her schedule and you are helping her, you are teaching, in the moment.

### Theme 3: Enhanced Center Staff Self-Care

Although teachers reported feeling well-supported by center leadership, they provided few concrete examples regarding center self-care policies and practices, instead making more general comments such as, “they checked-in,” “told us to take care of ourselves,” “always provided support,” or noted that the director’s office was “open for teachers who want to talk to her.” One teacher provided a concrete example of self-care policies in her program, including the provision of more frequent breaks and yoga classes for teachers:

You know, our school is doing always yoga for the teachers. They come into the classroom, and they asking us if we need a break. So, I feel in my case, in my school and my classroom is that I don’t have, I don’t feel like I’m overwhelmed with something. We have really good management that cares about you know how we feel, and so I think is, I cannot ask for more.

Directors noted that it was difficult for staff to attend additional professional development related to self-care on the weekends because they were so busy during the week and needed time off to relax. It should be noted that respondents often seemed to misinterpret and lack understanding of what was meant by self-care. For example, when asked about self-care, some provided responses related to school safety practices or how leadership provided teachers with information about COVID procedures. One teacher noted, “They really step up… guide us how we’re supposed to do and tell us about the test… about the quarantine, so I feel like they work so good with the teachers that we have that we didn’t have those problems.”

### Theme 4: Balancing in-person Interaction with Planned Virtual Delivery

Directors expressed mixed preference for the use of teleconsultations compared to in-person consultations. Teachers acknowledged the usefulness of technology for the convenience of consultation (particularly when time is limited) but indicated that they preferred in-person services. One teacher said, “Only thing is the convenience when it comes to virtual, it’s convenient, you know, versus something being in person.” Another noted, “I like to talk to person. You know like right now, I’m getting nervous because you’re on the screen. In person is easier for me to communicate…I’m not that good with technology.” Overall, teachers’ comments signaled a need for support and training with using virtual technology.

### Theme 5: Use of Existing Smartphone Applications for Parent Communication

Directors’ and teachers’ comments signaled the use of existing applications (e.g., Brightwheel™, ClassDojo©) to communicate with families around lesson plans. Teachers reported that they felt well-versed in and enjoyed using these apps: “Yes, because that helps the parents to know how the children are doing, what they do during the day, what they choose, and well the truth is that it stimulates them a lot…” They also indicated a need for an app that was potentially broader in scope and covered areas such as self-care for staff, families, and children.

Parents appeared to have the most interest in the use of mobile phone applications to support constant communication between themselves and their teachers:

Let me say the daily activities and routines of the of the toddlers and what they’re working on. I would definitely like to know that, so if I know what you’re working on in school, when you come home, I can reiterate and practice those same things.

They indicated an interest in having an app to support communication around safety (e.g., emergencies related to domestic or gun violence). They also described how an app could provide a platform for interfacing with other parents, provide them with tips and suggestions related to their child’s behavioral issues, and support mental health, nutrition, and physical activities outside of the existing early care and education center curriculum.

## Discussion

This qualitative study aimed to understand the perspectives of ethnically and racially diverse early care and education center directors, teachers, and parents regarding the impact of COVID-19 on early childhood education and inform the adaptation of a virtual ECMHC model. Our team used a rapid qualitative analysis to swiftly generate four themes related to participants’ experiences with COVID-19 and five themes related to their ongoing needs and suggestions for the implementation of our early childhood mental health consultation model. Participants’ comments suggested that COVID-19 exacerbated existing financial and infrastructure challenges and highlighted the need for programs to adapt center safety polices, classroom practices, and parent communication strategies, maintain good communication with and provide support for staff, and partner with families. They communicated that ongoing needs include increased financial support, outside behavioral support, and enhanced center staff self-care; they also suggested that our ECMHC model balance in-person interaction with planned virtual delivery and include the use of existing smartphone applications for communication with parents. Overall, this study provides valuable information for adapting ECMHC programs to meet the needs of early care and education centers serving primarily low-income, ethnic and racial minority families in the aftermath of COVID-19.

Consistent with previous literature, findings from this study highlight the consequences of COVID-19 and its associated mitigation efforts on early care and education, including its emotional and ongoing financial toll on the workforce (e.g., Crouse et al., 2023; Hoffman & Poll, 2022). Data from the RAPID Assessment of Pandemic Impact on Development-Early Childhood (RAPID-EC, 2023a) indicate that 68% of early care and education providers reported receiving relief funds from the American Rescue Plan Act (ARPA), noting that payroll expenses, both to pay current employees and raise their wages, were the most common use for the funds. This funding ended in September 2023. Despite the potential availability of these funds while our data were collected (between March and April 2023), participants consistently described the need for increased financial support for center operations and teachers’ wages. As recently as August 2023, 44% of U.S. early care and education providers, including center directors and teachers, surveyed in the RAPID-EC (2023b) reported experiencing at least one form of material hardship, with over a quarter reporting difficulty paying for utilities (28%) and food (26%). Given evidence on the impact of provider stress on young children’s social-emotional learning (Jeon et al., 2019), an important consideration for ECMHC programs around the country is the financial stability and viability of centers and their staff.

Our own ECMHC program, JS + CS, targets four pillars (safety planning, effective communication, adult self-care, and trauma-informed behavior support) based on Caring for our Children-National Health and Safety Standards (American Academy of Pediatrics, 2019) and supplemented with CDC COVID-19 guidelines for childcare centers (Centers for Disease Control and Prevention, 2022) and other evidence-based practices (e.g., Hemmeter et al., 2016). These pillars are consistent with a holistic integrative approach to child development and education, or one that focuses on nurturing the whole child (e.g., physical, emotional, social, cognitive) to foster sustained emotional and physical well-being (Rosen et al., 2020). Notably, although our safety planning pillar initially focused on physical and psychological safety (e.g., compliance with CDC disaster recovery guidelines; Natale et al., 2023), we have since expanded this definition to address the “financial safety” of centers. As part of our adapted program, we will be working to link centers to existing community resources (e.g., salary supplement award programs) and agencies that provide support to childcare centers to ensure their financial solvency. We are also providing centers with grant opportunities so that they can better support their programs and teachers.

Our results additionally highlight the importance of good communication between parents and early care and education staff. Open and honest communication is a vital mechanism to improve partnerships between parents and centers and increase parent engagement in their children’s development, leading to better child outcomes (Ansari & Gershoff, 2016; Rous et al., 2003). In this study, parents expressed appreciation for frequent communication from teachers about their children and classroom activities. They also expressed regret regarding the pandemic policies that prevented them from entering classrooms and the emotional impact of this loss of contact. It will be important for early care and education programs to increase parental involvement while maintaining safety procedures. To address this, our ECMHC program will train center staff to implement four virtual workshops with parents, one for each pillar of our model. The workshops will focus on building parents’ knowledge and efficacy for engaging in activities that support their children’s social and emotional development.

Interestingly, teachers in our study provided few examples regarding behavioral and social-emotional strategies they implemented in their classrooms. Behavior support strategies can reduce challenging behavior in young children, thus decreasing rates of expulsion or suspension. Challenging behaviors are a significant source of stress for early care and education teachers (Clayback & Williford, 2022), who are often undertrained and lack resources for effectively managing challenging behaviors. Lack of training and resources may lead to high teacher turnover rates given the imbalance between resources necessary to meet job demands (Schaack et al., 2022). Our program is adapting its approach to include the term “challenging behaviors” in the ECMHC materials used with teachers to help them make the connection that behavior support strategies prevent children’s challenging behavior and may thereby reduce their own stress.

Findings from our study also highlight the importance of supporting the mental health of early care and education staff and the need for ECMHC programs, particularly those serving ethnic and racial minorities, to enhance their provision of adult self-care. In a recent systematic review of early childhood educators’ burnout, authors found that low social capital, poor health, and lower wages were associated with increased risk for burnout while coaching, reflection and counseling-based interventions lowered risk for burnout (Ng et al., 2023). Interestingly, some participants in our study seemed to have difficulty responding to questions about “self-care,” which indicate that this term and its current conceptualizations may lack cultural and contextual relevance for those from less privileged backgrounds (Barks et al., 2023). To promote staff resilience, ECMHC programs serving racially and ethnically diverse populations should therefore focus on developing well-rounded and culturally-relevant wellness policies that provide staff with social support and stress management skills, such as scheduled stress check-ins with teachers and resiliency plans. Resiliency plans guide early care and learning staff to identify common physical, emotional, behavioral, cognitive, and social stress reactions and activities to address these reactions to support their well-being. Overall, addressing the well-being of early care and education staff is critically important given the link between teachers’ mental health, children’s mental health (Harding et al., 2019) and reductions in expulsions (Silver & Zinsser, 2020).

Regarding the provision of virtual support in the context of ECMHC, our findings suggest programs may need to balance in-person interaction with virtual delivery. Both center directors and teachers in our study expressed mixed preference for the use of teleconsultations compared to in-person consultations. An important lesson is the need to assess these preferences at the outset and potentially adapt the planned mode of delivery. At the beginning of our ongoing trial, for example, centers experienced challenges learning how to use Kubi telepresence robots (Jent et al. 2024), highlighting the importance of training, resources, and support for early care and education teachers (Ford et al., 2021). Our initial training was thus largely conducted in person, which allowed mental health consultants to build rapport more quickly prior to conducting virtual classroom observations, teacher consultations, and parent workshops remotely. Notably, we leveraged the use of existing digital resources following this study to improve communication between programs and families and to deliver educational content via existing applications (Brightwheel™, ClassDojo ©) to save time and enhance the reach of mental health consultants.

This study has several notable limitations and strengths. Although participants were primarily sampled from a single county in the U.S., this county is large, diverse, and home to the 3rd largest school district in the U.S. We triangulated data across three different “levels” of participants (directors, teachers, parents) from 12 different early care and education centers to generate our themes (Thurmond, 2001). We used RQA to make timely adaptations to our ECMHC model and employed recommended procedures for assuring quality and rigor throughout the process (St. George et al., 2023). RQA is increasingly being utilized in implementation science given its value and utility in guiding intervention adaptation, implementation, and dissemination efforts (Nevedal et al., 2021). Our study demonstrates the utility of this approach for informing adaptations to an ECMHC model during/post-COVID-19. Several of our adaptations, namely the reconceptualization of “safety planning” to include “financial safety”, the focus on virtual parent involvement, and the adaptation of behavior support materials will likely be critical to the long-term implementation and sustainability of the program.

## Conclusion

The impacts of COVID-19 have yet to be fully explored, especially within the already fragile early care and education system. It is imperative that we utilize effective strategies to strengthen the capacity of early care and education centers to respond to, care for, and protect our children. In the aftermath of COVID-19, ECHMC programs like JS + CS can build resilience in and buffer against the negative impacts of the pandemic on children, teachers, and centers. In the long-term, ECMHC can help build resilient behaviors that have life-long benefits. Overall, this study provides important insights regarding the adaptation of a virtual ECMHC program for racially and ethnically diverse populations of young children in early care and education programs. Although the federal COVID-19 public health emergency declaration ended in May 2023, the early care and education industry continues to experience challenges that pre-dated and were exacerbated by this pandemic. To address ongoing challenges and prepare for similar situations in the future, it is imperative that the field continues to systematically identify the downstream impacts of the pandemic and implement approaches that best meet the needs of diverse populations.
